# Cardiac Echinococcosis With Hepatic Involvement in a Child: A Case Report

**DOI:** 10.7759/cureus.30390

**Published:** 2022-10-17

**Authors:** Yassine Akrim, Fatima Babokh, Awatif El Hakkouni

**Affiliations:** 1 Department of Biology, Mohammed VI University Hospital, Cadi Ayyad University, Marrakech, MAR

**Keywords:** hydatidosis, echinococcosis, echinococcus granulosus, zoonotic infection, parasitic disease, hepatic hydatidosis, cardiac hydatidosis, hepato-cardiac hydatid cysts, hydatid cyst

## Abstract

Hydatidosis is endemic in Morocco. Cardiac localization of hydatid disease is a rare entity. Involvement of the interventricular septum is even rarer. We report the case of a 6-year-old girl with combined hepatocardiac hydatid disease. She was admitted with complaints of dyspnea, asthenia and vomiting. Ultrasound imaging and CT scan showed cystic lesions in the interventricular septum and in the liver. Serologic test results were positive. According to the biological and radiological findings, the diagnosis of echinococcosis with cardiac and hepatic involvement was suggested. Complete excision of the cardiac cyst was performed followed by anthelminthic treatment with albendazole as a supportive therapy. The confirmative diagnosis of hydatid disease was made by microscopic examination of the removed material. Our patient was referred to the department of general surgery to treat the liver lesions in the future. The postoperative period was unremarkable.

## Introduction

Hydatidosis is engendered by infection with the larvae of *Echinococcus granulosus*, which forms cysts in any organ or tissue of humans.* Echinococcus granulosus* tapeworms live in the intestines of dogs. Eggs are released in their faeces. Humans are accidental hosts and can be infected by ingesting food or water contaminated with stool from infected dogs.

Hydatidosis is an endemic disease in sheep-raising countries such as Morocco. It can involve any organ. Lungs and the liver are the most frequently affected sites [1]. Cardiac localization of hydatid disease is uncommon, and involvement of the interventricular septum is extremely rare [2].

We describe in this work the case of a 6-year-old girl with combined hepato-cardiac hydatid disease, who presented with complaints of dyspnea, weakness, and unexplained weight loss with progressive worsening.

## Case presentation

A 6-year-old girl from a rural area was referred to our hospital with asthenia, vomiting and difficulty in breathing for 10 months. In her medical history, a loss of weight and appetite was noticed by her family. During the clinical examination, the patient was conscious. The temperature, pulse rate, oxygen saturation, and blood pressure were normal. On abdominal examination, there was tenderness in the right hypochondriac region with moderate hepatomegaly. A review of other systems was unremarkable, and no abnormality in the cardiovascular auscultation was noted. The electrocardiogram showed a heart rate of 70 bpm with a normal sinus rhythm. Laboratory investigations revealed a normal level of hemoglobin (13.5 g/dl), white blood cells (9,600 cells/mm3), and C-reactive protein (5 mg/l). The other tests (liver function, levels of electrolytes, creatinine, urea, and coagulation tests) were within the normal ranges. Abdominal ultrasound detected multiple liver cysts without calcifications.

**Figure 1:**
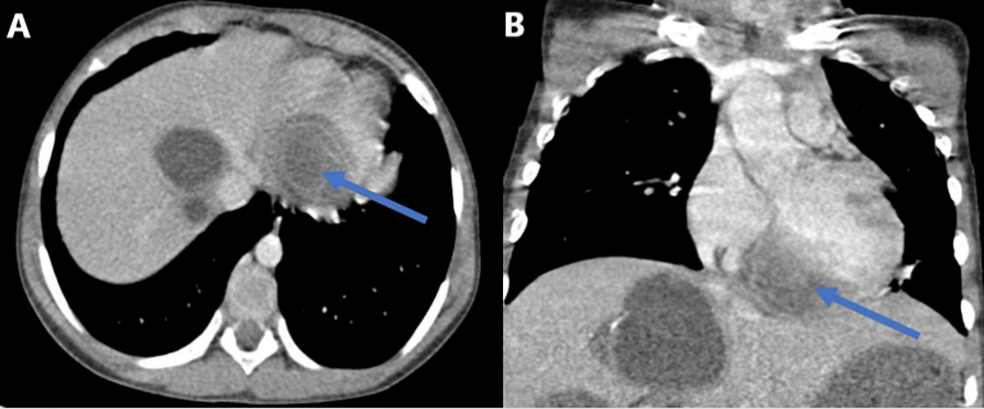
Axial (A) and coronal (B) computed tomography showing the cystic lesion in the interventricular septum (arrows).

**Figure 2:**
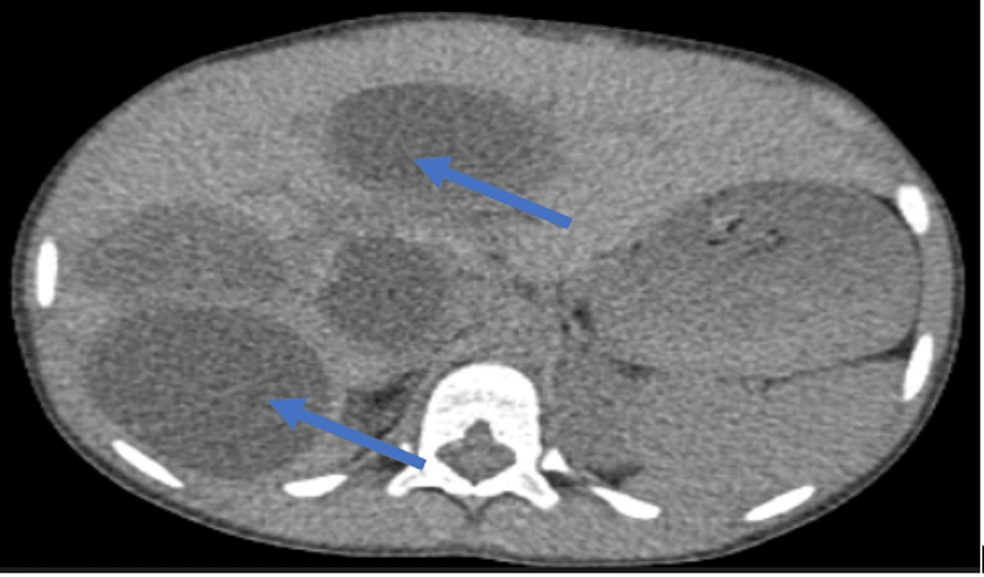
CT scan showing the axial image of multiple hydatid cysts in the liver (arrows).

Computed tomographic images of the chest showed cystic formation measuring 5.5 × 4.5 cm in size located in the interventricular septum (Figure 1). On abdominal computed tomography, multiple hepatic cysts of variable sizes were observed. The largest cyst was 7 cm × 6 cm in size (Figure 2).

Transthoracic echocardiography confirmed a well-defined cyst mass in the interventricular septum with a normal ejection fraction. Serologic test results for hydatidosis were positive. Thus, the diagnosis of hydatid disease with cardiac and hepatic involvement was confirmed.

**Figure 3:**
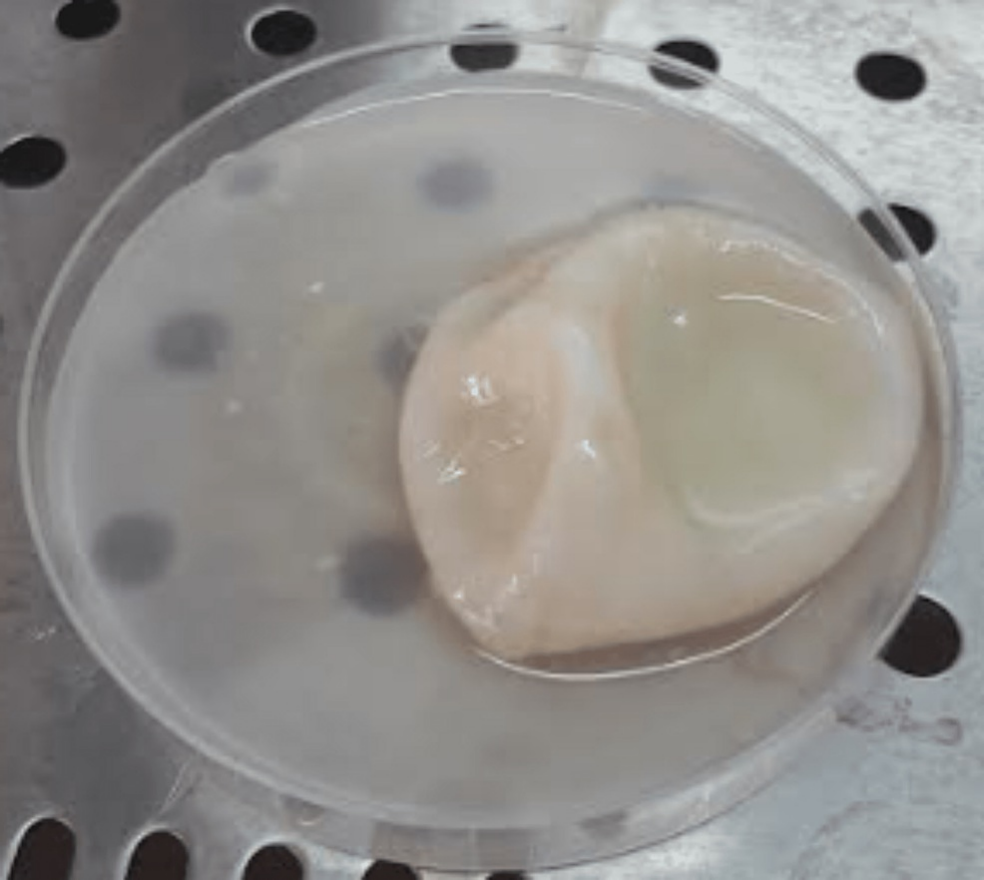
Macroscopic findings of the excised hydatid cyst.

**Figure 4:**
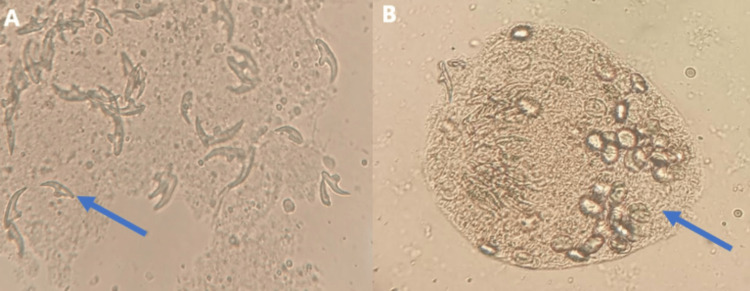
Direct microscopic examination (40x magnification) showing free hooklets (A) and protoscoleces (B) of Echinococcus.

Upon surgical exploration, drainage of viscous fluid from the cyst with radical cystectomy was performed. The surgical specimen was sent to the Parasitology Laboratory for analysis. The macroscopic examination of the removed material showed a white, pearly, spherical cyst containing clear fluid (Figure 3). Microscopic analysis revealed several protoscoleces with free hooklets of *Echinococcus* (Figure 4).

Antihelmenthic treatment with albendazole was started with regular control of liver function tests and blood counts. The patient was discharged after two weeks and was referred to the department of general surgery to treat the liver lesions in the future. The postoperative period was unremarkable. Three months after the surgery, the patient remained asymptomatic.

## Discussion

Hydatidosis is actually endemic in Morocco and is considered a significant health problem. Hydatid disease usually involves the liver and lungs. The first case of cardiac hydatidosis was described by Williams in 1936 as a rare entity [3]. The most involved location is the left ventricle. Hydatid cyst over the interventricular septum is even rarer [4]. Frequently, cardiac hydatidosis is associated with multiple organ involvement. Our patient had a combination of hepatic and cardiac hydatid cysts.

Hydatidosis is caused by infection with the larvae of the tapeworm *Echinococcus granulosus*. Humans are infected by consuming food or water contaminated with echinococcus eggs from the feces of dogs [5].

Due to the slow growth of hydatid cysts (about 1 cm per year), patients with cardiac hydatidosis usually remain asymptomatic, and only 10% present clinical signs [6]. The clinical presentation differs in accordance with the localization, volume, and number of cysts. Chest pain, shortness of breath, cough, and palpitations are the most common symptoms of cardiac hydatid cysts [7]. Cardiac echinococcosis can cause serious complications, such as anaphylactic reactions, valvular dysfunction, cardiac tamponade and sudden cardiac death [8]. Our patient presented with difficulty in breathing, vomiting, and asthenia.

Echocardiography is used as the first-line imaging technique to show the number, size, localization and also hemodynamic effect of the cysts. Differential diagnosis includes cardiac tumors, metastasis and interventricular septum aneurysm. Other modalities like computed tomography and MRI are useful to establish the diagnosis, study the relationship with adjacent tissues and help in planning the treatment [9]. Our radiological findings, in this case, showed a well-defined cystic lesion localized in the interventricular septum with multiple hepatic cysts of variable sizes.

Concerning biological investigations, ELISA (enzyme-linked immunoassay) method has a high sensitivity for hydatidosis. Due to other parasitic diseases, we can have a false positive by cross-reactions. ELISA is usually followed by Western blot as a confirmatory and specific test [10].

In the case of cardiac hydatid disease, surgical excision is the treatment of choice to avoid mortal complications. Anthelminthic therapy with albendazole must be used adjunctively in order to prevent a recurrence [11]. Exclusive medical treatment is administered to manage small-sized or calcified lesions or inoperable forms [12].

## Conclusions

Even in endemic regions, cardiac echinococcosis is a rare entity. Symptoms of cardiac hydatidosis can vary in severity. The clinical presentation may range from asymptomatic form to sudden death. Imaging techniques are mandatory for early detection of the disease. Cardiac hydatidosis must be suspected in patients from endemic areas presenting cardiac cystic tumors. Surgical excision of the cyst without rupture is the modality of choice. Supplemental treatment with albendazole is strongly recommended in order to decrease the risk of recurrence. Prevention initiatives at local and national level are the best way to decrease the prevalence of hydatidosis.

## References

[1] Krige JE, Beckingham IJ (2001). ABC of diseases of liver, pancreas, and biliary system. BMJ.

[2] Shojaei E, Yassin Z, Rezahosseini O (2016). Cardiac hydatid cyst: a case report. Iran J Public Health.

[3] Oner T, Korun O, Celebi A (2019). A cardiac hydatid cyst mimicking a pericardial tumour in a paediatric case. Cardiol Young.

[4] Oraha AY, Faqe DA, Kadoura M, Kakamad FH, Yaldo FF, Aziz SQ (2018). Cardiac hydatid cysts; presentation and management. A case series. Ann Med Surg (Lond).

[5] Vlad DC, Neghina AM, Dumitrascu V, Marincu I, Neghina R, Calma CL (2013). Cystic echinococcosis in children and adults: a seven-year comparative study in western Romania. Foodborne Pathog Dis.

[6] Yaliniz H, Tokcan A, Salih OK, Ulus T (2006). Surgical treatment of cardiac hydatid disease: a report of 7 cases. Tex Heart Inst J.

[7] Fennira S, Kamoun S, Besbes B (2019). Cardiac hydatid cyst in the interventricular septum: a literature review. Int J Infect Dis.

[8] Pedrosa I, Saíz A, Arrazola J, Ferreirós J, Pedrosa CS (2000). Hydatid disease: radiologic and pathologic features and complications. Radiographics.

[9] Kankilic N, Aydin MS, Günendi T, Göz M (2020). Unusual hydatid cysts: cardiac and pelvic-ilio femoral hydatid cyst case reports and literature review. Braz J Cardiovasc Surg.

[10] Schantz PM, Gottstein B (1986). Echinococcosis (hydatidosis). Immunodiagnosis of Parasitic Diseases, Volume 1.

[11] Brunetti E, Kern P, Vuitton DA (2010). Expert consensus for the diagnosis and treatment of cystic and alveolar echinococcosis in humans. Acta Trop.

[12] Bozbuga N, Erentug V, Akinci E, Yakut C (2013). Is surgical therapy the only treatment of choice for cardiac echinococcosis with multiple organ involvement?. Interact Cardiovasc Thorac Surg.

